# BDNF-TrkB Pathway Mediates Neuroprotection of Hydrogen Sulfide against Formaldehyde-Induced Toxicity to PC12 Cells

**DOI:** 10.1371/journal.pone.0119478

**Published:** 2015-03-06

**Authors:** Jia-Mei Jiang, Cheng-Fang Zhou, Sheng-Lan Gao, Ying Tian, Chun-Yan Wang, Li Wang, Hong-Feng Gu, Xiao-Qing Tang

**Affiliations:** 1 Department of Physiology & Institute of Neuroscience, Medical College, University of South China, Hengyang, 42100, Hunan, P. R. China; 2 Key Laboratory for Cognitive Disorders and Neurodegenerative Diseases, University of South China, Hengyang, 421001, Hunan, P. R. China; 3 Department of Biochemistry, Medical College, University of South China, Hengyang, 421001, Hunan, P.R. China; 4 Department of Pathophysiology, Medical College, University of South China, Hengyang, 421001, Hunan, P.R. China; 5 Department of Anthropotomy, Medical College, University of South China, Hengyang, 421001, Hunan, P.R. China; Indian Institute of Toxicology Reserach, INDIA

## Abstract

Formaldehyde (FA) is a common environmental contaminant that has toxic effects on the central nervous system (CNS). Our previous data demonstrated that hydrogen sulfide (H_2_S), the third endogenous gaseous mediator, has protective effects against FA-induced neurotoxicity. As is known to all, Brain-derived neurotropic factor (BDNF), a member of the neurotrophin gene family, mediates its neuroprotective properties via various intracellular signaling pathways triggered by activating the tyrosine kinase receptor B (TrkB). Intriguingly, our previous data have illustrated the upregulatory role of H_2_S on BDNF protein expression in the hippocampus of rats. Therefore, in this study, we hypothesized that H_2_S provides neuroprotection against FA toxicity by regulating BDNF-TrkB pathway. In the present study, we found that NaHS, a donor of H_2_S, upregulated the level of BDNF protein in PC12 cells, and significantly rescued FA-induced downregulation of BDNF levels. Furthermore, we found that pretreatment of PC12 cells with K252a, an inhibitor of the BDNF receptor TrkB, markedly reversed the inhibition of NaHS on FA-induced cytotoxicity and ablated the protective effects of NaHS on FA-induced oxidative stress, including the accumulation of intracellular reactive oxygen species (ROS), 4-hydroxy-2-trans-nonenal (4-HNE), and malondialdehyde (MDA). We also showed that K252a abolished the inhibition of NaHS on FA-induced apoptosis, as well as the activation of caspase-3 in PC12 cells. In addition, K252a reversed the protection of H_2_S against FA-induced downregulation of Bcl-2 protein expression and upregulation of Bax protein expression in PC12 cells. These data indicate that the BDNF-TrkB pathway mediates the neuroprotection of H_2_S against FA-induced cytotoxicity, oxidative stress and apoptosis in PC12 cells. These findings provide a novel mechanism underlying the protection of H_2_S against FA-induced neurotoxicity.

## Introduction

Formaldehyde (FA), a common environmental contaminant, is widely found in domestic air, tobacco smoke, garments, paint, and industrial and medical products [1,2]. Increasing evidence indicated that FA is toxic to mammals [3–6], especially inducing impairment in learning and memory as well as neurotoxicity in the central nervous system (CNS) [7–10]. Epidemiological data showed that long-term exposure to FA causes neurocognitive and neurobehavioral impairment in histology technicians and workers [11]. In several experimental models, it has been shown that FA exposure induces the apoptosis and neurotoxicity in the cultured cortical neurons and PC12 cells [12,13], and elicits behavioral and learning and memory disorders in rats and mice[8,9]. Although a lot of literature describes the neurotoxicity of FA, there is no effective way to defend FA-induced neurotoxicity. Thus, it is important to explore novel therapeutic targets for the neurotoxicity of FA.

Hydrogen sulfide (H_2_S) is recognized as the third ‘gasotransmitter’ alongside nitric oxide (NO) and carbon monoxide (CO) [14,15]. Expanding evidence documented that H_2_S, at physiological concentrations (50–160 mmol/L) in brain, is a novel neuroprotective agents [16–19]. Many studies have confirmed that H_2_S can protect neurons against oxidative stress, apoptosis, and endocytoplasmic reticulum (ER) stress impairment induced by multiple reagents [20–23]. Interestingly, our previous data demonstrated that FA exposures downregulates the production of endogenous H_2_S in PC12 cell and in the hippocampus of rats [24,25]. Thus, it is worth thinking whether increasing the levels of H_2_S can inhibit FA-induced neurotoxicity. Our recent data showed that NaHS, an H_2_S donor, protects PC12 cells against FA-induced endoplasmic reticulum stress, mitochondrial dysfunction and apoptosis [26,27]. These data demonstrate the protection of H_2_S against the neurotoxicity of FA and suggest a promising future of H_2_S-based preventions for FA-induced neurotoxicity. However, the potential mechanisms underlying the protection of H_2_S against FA-induced neurotoxicity are largely unknown.

Brain-derived neurotrophic factor (BDNF), a member of the neurotrophin family, exerts its roles via its high affinity receptor tyrosine protein kinase B (TrkB) [28]. BDNF has been shown to rescue neuronal cells from neurodegeneration owing to injuries in the CNS [29–33] and prevent oxidative damage in many cultivated neurons [34–36]. Boyadjieva NI and his colleague demonstrated that BDNF downregulates the ethanol-induced cellular oxidative stress and apoptosis in developing hypothalamic neuronal cells [37]. Furthermore, our previous study proved that BDNF-TrkB pathway contributes to the protection of H_2_S against homocysteine-induced ER stress and neuronal apoptosis in hippocampus of rat [38]. Therefore, this work was designed to investigate whether the BDNF-TrkB pathway also mediates the protection of H_2_S against FA-induced cytotoxicity, oxidative stress, and apoptosis in PC12 cells.

The present studies examine the role of BDNF-TrkB pathway in the neuroprotective properties of H_2_S against FA-induced toxicity in PC12 cells. We demonstrated that NaHS, a donor of H_2_S, significantly rescues FA-induced the downregulation of BDNF expression in PC12 cells and that K252a, a BDNF-TrkB pathway inhibitor, abolished the protective effects of H_2_S against FA-induced cytotoxicity, oxidative stress, and apoptosis. Our data indicate that BDNF-TrkB pathway mediates the protective role of H_2_S against FA-induced neurotoxicity.

## Materials and Methods

### Reagent

Formaldehyde (FA), Sodium hydrosulfide (NaHS, a donor of H_2_S), K252a (a selective pharmacological pan-Trk inhibitor) and nitro blue tetrazolium (NBT) were supplied by Sigma Chemical CO (St. Louis, MO, USA). Cell counting kit-8 (CCK-8) was purchased from Dojindo Molecular Technologies, Inc. (Rockvile, MD, USA). Specific monoclonal antibody to BDNF was obtained from Epitomic Inc (Burlingame, UK). Specific monoclonal antibody to Bax was purchased from Abcam Technology (Cambridge, CB, UK) and Specific monoclonal antibody to Bcl-2 was purchased from Cell Signaling Technology, Inc (Beverly, MA, USA). Beta-actin antibody, Goat anti-mouse antibody, Goat anti-rabbit antibody, and Goat anti-Rat antibody were purchased from Proteintech (Danvers, MA, USA). Caspase-3, 4-hydroxy-2-trans-nonenal (4-HNE), and malondialdehyde (MDA) enzyme-linked immunosorbent assay (ELISA) Kits were bought from USCN Life Science Inc (Wuhan, Hubei, China). Bicinchoninic Acid (BCA) Protein Assay Kit was obtained from Beyotime Institute of Biotechnology (Shanghai, China)

### Cell culture

The PC12 cell line was derived from rat pheochromocytoma, a tumor arising of the adrenal medulla [39] and represents a valuable model to study cell fate such as neuronal differentiation, cell proliferation, or cell survival [40,41]. PC12 cells were (ATCC, CRL-1721) generously provided by the Sun Yat-sen University Experimental Animal Center (Guangzhou, China) and cultured in Dulbecco’s modified Eagle’s Medium (DMEM) containing 10% heat-inactivated fetal bovine serum (FBS) and 1% penicillin-streptomycin (PS) at 37°C under a humidified incubator with an atmosphere 5% CO_2_ and 95% air. Medium was replaced every 2 days.

### Determination of Cell Viability

The viability of PC12 cells was determined by CCK-8 assay according to the manufacturer's instructions [42]. PC12 cells were cultured in 96-well plates at 37°C under an atmosphere 5% CO_2_ and 95% air._._ At the end of treatment, CCK-8 reagent (5 μl) was added to each well of the plates and then the plates were incubated at 37°C for 3–4 h in the incubator. Absorbance at a wavelength of 450 nm was measured with a microplate reader (Molecular Devices, Sunnyvale, CA, USA). Means of 3–5 wells optical density (OD) in the indicated groups were used to calculate the cell viability that was expressed as a percentage of the cell survival rate compared with the control. All experiments were done in triplicate and repeated three independent times.

### Flow Cytometry Analysis of Cell Apoptosis

The apoptosis of PC12 cells was detected by propidium iodide (PI) staining. PC12 cells in logarithmic phase growth were seeded in 6-well plate with 10^6^ cells in each well. When the cells were about 70% confluent, PC12 cells were administered with indicated conditioned-mediums for 24 h. After exposure terminated, the medium was removed and the cells were rinsed with PBS. Each group of cells was harvested and centrifuged at 250 *g* for 10 min. Cells were washed twice with PBS and fixed with 70% pre-refrigerated ethanol for 24 h at −20°C. After washing the cells with PBS twice, 1 mg/mL RNase (Sigma Chemical Co., St. Louis, MO) was added and incubated for 30 min at 37°C. Then, the cells were stained with PI (at a final concentration of 50 mg/L) in the dark at 4°C for 30 min before flow cytometric (FCM, Beckman-Coulter, Miami, FL, USA) analysis. In the DNA histogram, the amplitude of the sub-G1 DNA peak represents the amount of apoptotic cells. Experiments were repeated three times independently.

### Measurement of Intracellular reactive oxygen species (ROS) Generation

Intracellular ROS were measured by the nitroblue tetrazolium (NBT) test which is converted to purple formazan by superoxide anion [43]. Briefly, the PC12 cells (1 x 10^5^ cells per well) were plated in 96-well tissue culture plates overnight. Conditioned-mediums were administered as indicated for 24 h. After removal of the supernatant, the cells were washed with PBS and then 100 μL NBT (1.0 mg/mL in DMEM) was added to each well. After incubation at 37°C for 2 h, the cells were washed with PBS and 100 μL KOH (2 mol/l) and 100 μL dimethyl sulfoxide (DMSO) were added to dissolve the cells. The absorbance at 570 nm was determined using a microplate reader. Experiments were repeated three times independently.

### ELISA for caspase-3 activity

The activity of caspase-3 was determined by caspase-3 activity kit according to manufacturer instructions. In brief, at the end of treatment, cells were harvested and split by Ultrasonic Cell Disruption System (5s, 15 times, 4°C). The homogenized samples were then centrifuged at 5000 g for 20 min and the supernatant protein concentration was quantified by BCA protein assay kit. 100 μl of diluted samples were mixed in a white-walled 96-well which was coated with an antibody specific for caspase-3 and incubated at 37°C for 2 h. Removing the liquid of each well, 100 μl of reaction buffer A and B were added to the microplate respectively. Subsequently, 90 μl aliquot of caspase-3 reagent was added to each well and fluorescence was measured at 450 nm with a microplate reader. Experiments were repeated three times independently.

### ELISAS for MDA and 4-HNE

At the end of treatment, cells were collected with ice-cold PBS and homogenized with Ultrasonic Cell Disruption System. The homogenate of cells was centrifuged at 5000 *g* for 20 min and the supernatant was collected. The protein concentration was determined with BCA Protein Assay Kit. The formation of lipid peroxidation in cells was measured using MDA and 4-HNE ELISA Kits. Briefly, protein sample (10 μg/ ml, 50 μl) was added to the 96-well protein binding plate and incubated at 37°C for 2 h, and then washed two times with PBS. 100 μl of diluent per well was added and incubated for 2 h at room temperature on an orbital shaker, and then washed three times with wash buffer with thorough aspiration between each wash. The diluted anti-MDA (50 μl) or anti-4-HNE antibody (50 μl) was added to all wells and incubate for 1 h at room temperature on an orbital shaker, and then washed three times with wash buffer. Subsequently, the diluted secondary antibody- HRP conjugate (50 μl) was added to all wells and incubate for 1 h at room temperature on an orbital shaker. Substrate solution (50 μl) was then added to each well and incubated for 2–30 min at room temperature on an orbital shaker. If color changes rapidly, the reaction was stopped by adding 50 μl of stop solution. The absorbance of each well was read on a microplate reader at 450 nm. Experiments were repeated three times independently.

### Western blot analysis for the levels of BDNF, Bcl-2, and Bax protein expression

Cell lysates were used to examine the expressions of BDNF, Bcl-2 and Bax protein. Logarithmic phase PC12 cells were seeded at a concentration of 10^6^ cells per well on 6-well plates. At the end of treatment, cells were washed with 4°C PBS and then lysed in an ice-cold lysis buffer [20 mM Tris–HCl, pH 7.5, 150 mM NaCl, 1% Triton X-100, 1 mM phenylmethylsulphonyl fluoride (PMSF), 1 mM Na_3_VO_4_, leupeptin, and EDTA] for 30 min. Soluble fractions were collected following centrifugation for 10 min at 12,000 rpm and were stored at −80°C until used. The protein concentration was determined by BCA Protein Assay Kit. An equal amount of 30–50 μg of proteins was separated by 8–12% sodium dodecyl sulfate-polyacrylamide gel (SDS-PAGE) and transferred to polyvinylidene fluoride (PVDF) membranes by electroblotting. Non-specific protein binding was blocked with 5% non-fat dried milk in TBST buffer (pH 7.6, 3.03g Tris base, 18.8g glycocine, 1g SDS, 1000 ml ddH2O, plus 1ml Tween-20) for 2 h at room temperature. Then, the membranes were incubated overnight at room temperature with diluted primary antibody: BDNF Rabbit Monoclonal Antibody (1:1000, EPITMICS, EP1293), Bcl-2 Antibody (1:500, Cell signaling, #2876), Mouse monoclonal to Bax (1:1000, abcam, ab5714). A monoclonal antibody against β-actin (1:2000, Proteintech, 60008–1-IG-16) was used as control for protein gel loading. The membranes were then washed three times with TBST, and incubated with HRP-conjugated secondary antibody (1:5000, Proteintech, SA00001–2) at room temperature for 2 h. Protein bands were analyzed using the enhanced chemiluminescence detection system (BeyoECL Plus kit, Beyotime, P0018). Integrated optical densities were analyzed using Image J software. Experiments were repeated three times independently.

### Statistical analysis

All experiments were repeated at least three times. Data are expressed as the mean ± S.E.M. Statistical significance is assessed using one-way analysis of variance (ANOVA) and Least-significant difference (LSD) test for post-hoc comparisons). Differences were considered significant at *P* < 0.05.

## Results

### H_2_S upregulates the level of BDNF protein in PC12 cells

**Fig 1:**
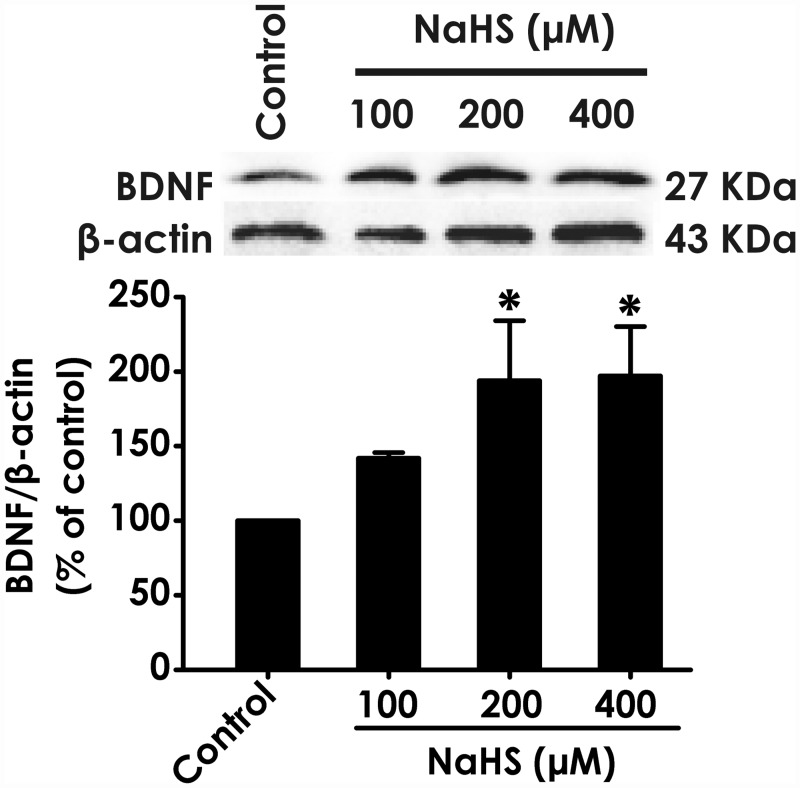
Effect of H_2_S on the expression of BDNF protein in PC12 cells. PC12 cells were treated with NaHS (100, 200, and 400 μM) for 24 h. The expression of BDNF protein was determined by Western blot using anti-BDNF antibody, and β-actin was used as a loading control. The ratio of BDNF to β-actin is normalized by the value in control group. Values are expressed as the mean ± S.E.M. of three independent experiments. *P < 0.05, versus control group.

To illustrate whether BDNF is involved in the protective effect of H_2_S against FA-elicited neurotoxicity, we first investigated the effects of H_2_S on the level of BDNF protein in PC12 cells. After treatment of PC12 cells with different concentrations of NaHS (100, 200, and 400mM), a donor of H_2_S, the level of BDNF protein in cells was markedly increased (Fig. 1). This data indicated that BDNF may be involved in the neuroprotection of H_2_S.

### H_2_S prevents formaldehyde-induced downregulation of BDNF in PC12 cells

**Fig 2:**
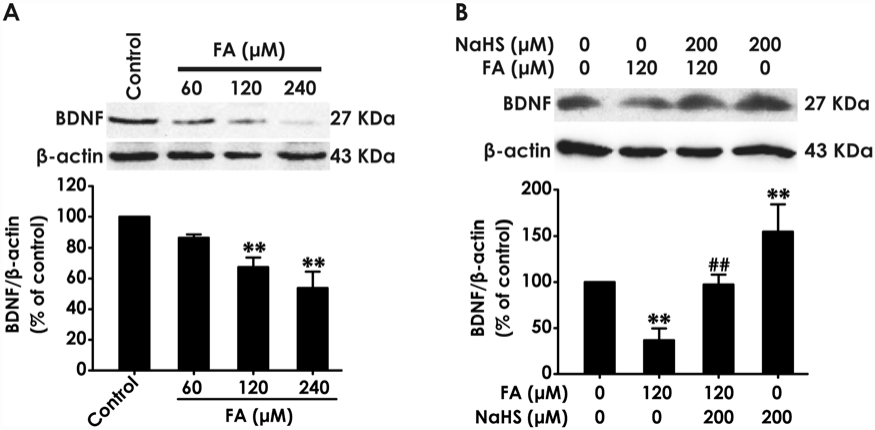
Effect of H_2_S on the expression of BDNF protein in formaldehyde-treated PC12 cells. (A) PC12 cells were treated with formaldehyde (FA, 60, 120, or 240 μM) for 24 h. (B)After pretreatment of PC12 cells with NaHS (200 μM) for 30 min, FA (120 μM) was added to culture medium and coincubated for 24 h. The expression of BDNF protein was determined by Western blot using anti-BDNF antibody, and β-actin was used as a loading control. The ratio of BDNF to β-actin is normalized by the value in control group. Values were expressed as the mean ± S.E.M. of three independent experiments. **P < 0.01, versus control group; ^##^ *P* < 0.01, versus FA-treated along group.

Next, we explored the effect of H_2_S on the expression of BDNF protein in formaldehyde (FA)-exposed PC12 cells. We found that treatment with different concentrations of FA (60, 120, or 240 μM, for 24 h) markedly downregulated the levels of BDNF in PC12 cells (Fig. 2A). Interestingly, pretreatment with NaHS (200 μM) for 30 min significantly rescued FA-induced the downregulation of BDNF protein in PC12 cells (Fig. 2B). In addition, treatment of PC12 cells with NaHS alone also upregulated the levels of BDNF protein. These data indicated that BDNF may be involved in the protection of H_2_S against FA-induced neurotoxicity.

### Blockage of BDNF-TrkB pathway reverses the protective effect of H_2_S against FA-indcued cytotoxicity in PC12 cells

**Fig 3:**
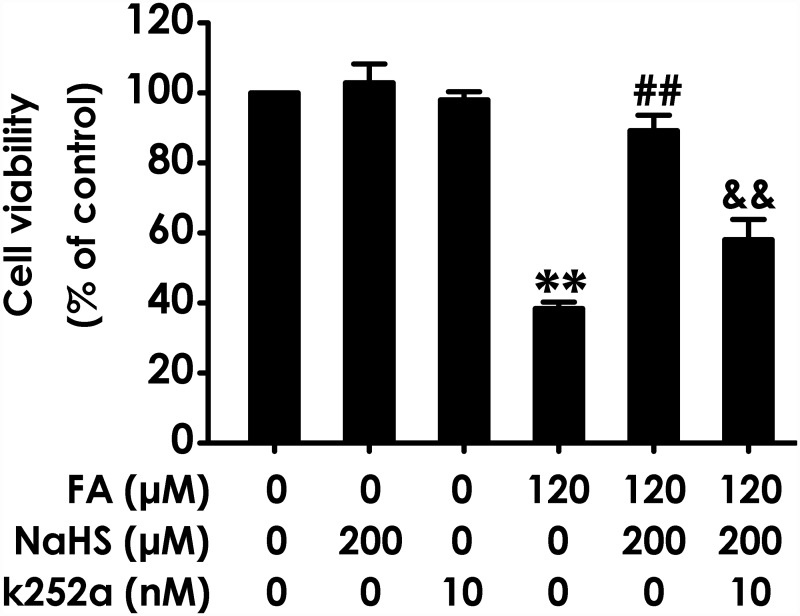
Effect of K252a on H_2_S-induced protection against formaldehyde-exerted cytotoxicity in PC12 cells. PC12 cells were preincubated with K252a (10 nM) for 30 min before pretreatment with NaHS (200 μM) for 30 min, and then cotreated with formaldehyde (FA, 120 μM) for 24 h. Cell viability was determined by the CCK-8 assay. Results were expressed as the mean ± S.E.M. of three independent experiments. **P < 0.01, versus control group; ^##^ *P* < 0.01, versus FA-treated alone group; ^&&^ *P* < 0.01, versus cotreatment with NaHS and FA group.

To confirm the hypothesis that BDNF-TrkB pathway mediates the protection of H_2_S against FA-induced neurotoxicity, we next explored whether K252a, a specific BDNF-TrkB pathway inhibitor, reverses the protective role of H_2_S against FA-indcued cytotoxicity. Pretreatment of PC12 cells with K252a (10 nM) for 30 min before the administration of NaHS (200 mM) significantly attenuated NaHS-suppressed the loss of cell viability induced by treatment with FA (120 μM) (Fig. 3). K252a (10 nM) or NaHS (200 mM) alone did not affect the viability of PC12 cells. These data suggested that H_2_S protects PC12 cells against FA-induced cytotoxicity via BDNF-TrkB pathway.

### Blocking BDNF-TrkB pathway prevents the inhibitiory effects of H_2_S against FA-induced oxidative stress

**Fig 4:**
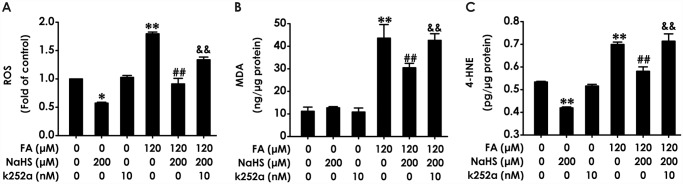
Effect of K252a on H_2_S-induced suppression in formaldehyde-mediated oxidative stress in PC12 cells. PC12 cells were preincubated with K252a (10 nM) for 30 min before pretreatment with NaHS (200 μM) for 30 min, and then cotreated with formaldehyde (FA, 120 μM) for 24 h. (A) The generation of intracellular ROS was measured by NBT reduction assay as described in “Materials and methods.”. (B) The level of MDA in PC12 cells was measured by MDA Elisa kits. (C) The level of 4-HNE in PC12 cells was measured by 4-HNE Elisa kits. Results were expressed as the mean ± S.E.M. of three independent experiments. *P < 0.05, **P < 0.01, versus control group; ^##^ *P* <0.01, versus FA-treated alone group; ^&&^ *P* < 0.01, versus cotreated with NaHS and FA group.

To further confirm the mediated role of BDNF-TrkB pathway in the protection of H_2_S against FA-induced neurotoxicity, we explored whether K252a reverses the protection of H_2_S against FA-induced oxidative stress in PC12 cells by detecting the levels of intracellular ROS, MDA, and 4-HNE. K252a (10 nM, for 24 h) alone had no influence on the levels of intracellular ROS, MDA, and 4-HNE in PC12 cells (Fig. 4A-C). However, pretreatment with K252a (10 nM) for 30 min markedly suppressed the inhibitiory effects of NaHS (200 μM) on the increases in the levels of intracellular ROS (Fig. 4A), MDA (Fig. 4B), and 4-HNE (Fig. 4C) in PC12 cells induced by treatment of 120 μM of FA for 24 h. Notably, treatment of PC12 cells with NaHS alone downregulated the levels of ROS (Fig. 4A) and 4-HNE (Fig. 4C). These data indicated that inhibition of BDNF-TrkB pathway reverses H_2_S-caused protection against FA-induced oxidative stress in PC12 cells.

### Inhibition of BDNF-TrkB reverses the protective effect of H_2_S against FA-induced apoptosis in PC12 cells

**Fig 5:**
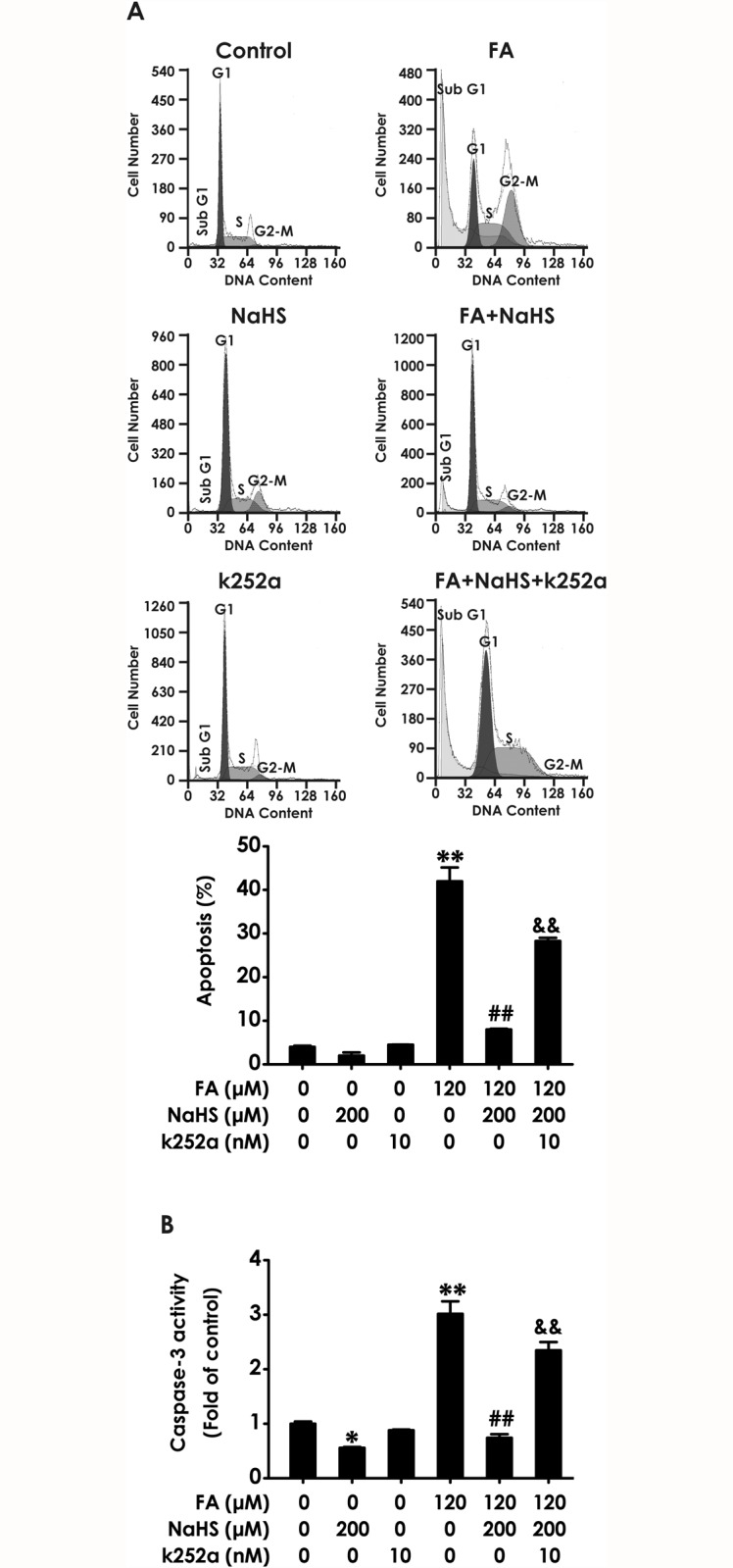
Effect of K252a on H_2_S-induced protection against formaldehyde-elicited apoptosis in PC12 cells. PC12 cells were preincubated with K252a (10 nM) for 30 min before pretreatment with NaHS (200 μM) for 30 min, and then cotreated with formaldehyde (FA, 120 μM) for 24 h. (A) The rate of apoptosis was assessed by flow cytometry after PI staining. (B) The activity of caspase-3 was determined by caspase-3 activity Elisa kit. Results were expressed as the mean ± S.E.M. of three independent experiments. *P < 0.05, **P < 0.01, versus control group; ^##^ *P* < 0.01, versus FA-treated alone group; ^&&^ *P* < 0.01, versus cotreated with NaHS and FA group.

We further investigated whether K252a reverses the protection of NaHS against FA-induced apoptosis. The statistical findings from FCM analysis after PI staining indicated that K252a reverses the protection of NaHS against FA-induced apoptosis. As shown in Fig. 5A, exposure of PC12 cells to FA (120 μM, for 24 h) caused significant apoptosis and the apoptotic effects induced by FA were inhibited by co-treatment with NaHS (200 μM) for 24 h; however, this protective effect of NaHS was markedly prevented by pretreatment with 10 nM of k252a for 30 min. Caspase-3 is a critical executioner of apoptosis. As shown in Fig. 5B, pretreatment with k252a (10 nM, for 30 min) significantly abolished NaHS (200 μM, for 24 h)-suppressed the increase in caspase-3 activity induced by treatment of 120 μM of FA for 24 h. In addition, the activity of caspase-3 was also decreased caused by NaHS alone (Fig. 5B), which was consistent with the protection of NaHS against FA-induced apoptosis. These data indicated that BDNF-TrkB pathway mediates H_2_S-caused protection against FA-induced apoptosis in PC12 cells.

### Inhibition of BDNF-TrkB reverses the protective effect of H_2_S against FA-induced modification of Bax and Bcl-2 in PC12 cells

**Fig 6:**
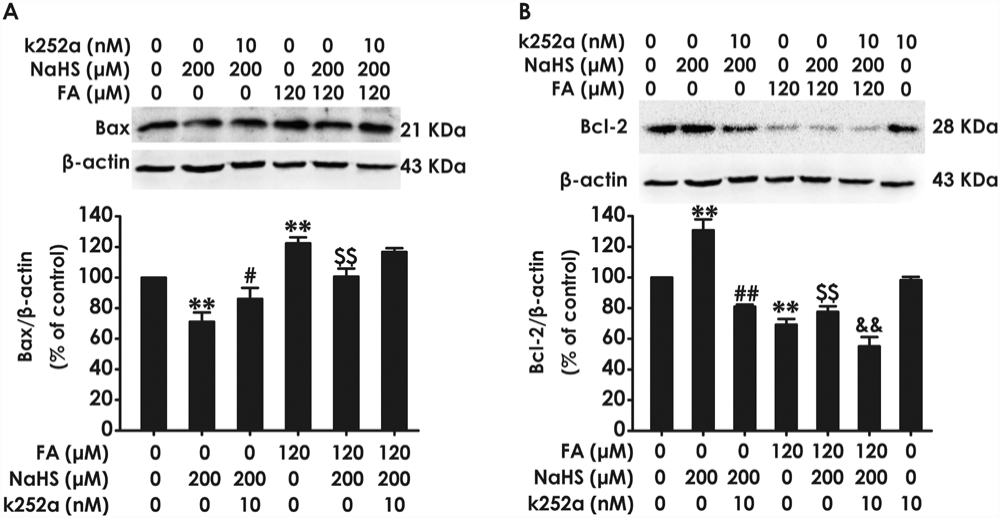
Effect of K252a on H_2_S-caused suppression in formaldehyde-induced upregulation of Bax and downregulation of Bcl-2 in PC12 cells. PC12 cells were pre-incubated with K252a (10 nM) for 30 min before pretreatment with NaHS (200 μM) for 30 min prior and the cotreated with formaldehyde (FA, 120 μM) for 24 h. The expression of Bax (A) and Bcl-2 (B) were detected by Western blot using anti-Bax antibody and anti-Bcl-2 antibody, respectively. In all blots, β-actin was used as a loading control. The ratio of Bax or Bcl-2 to β-actin was normalized by the value in control group. Values were expressed as the mean ± S.E.M. of three independent experiments. **P < 0.01, versus control group; ^##^ *P* <0.01, versus NaHS-treated alone group; ^$ $^ *P* <0.01, versus FA-treated alone group; ^&&^ *P* < 0.01, versus cotreated with NaHS and FA group.

Finally, we investigated whether BDNF-TrkB pathway mediates the protective effect of H_2_S against FA-induced change in apoptosis-related proteins in PC12 cells. We found that pretreatment of PC12 cells with K252a (10 nM, for 30 min) reverses the protection of NaHS against FA-induced upregulation of Bax protein expression (Fig. 6A) and downregulation of Bcl-2 protein expression (Fig. 6B). Notably, treatment with NaHS alone (200 μM, 24 h) decreased the levels of Bax (Fig. 6A) and increased the levels of Bcl-2 (Fig. 6B) in PC12 cells. However, co-treatment with K252a (10 nM) and NaHS(200 μM) for 24 h significantly abolished the NaHS-induced downregulation of Bax (Fig. 6A) and upregulation of Bcl-2 (Fig. 6B). These results indicated that BDNF-TrkB pathway is able to mediate the inhibitory role of H_2_S in FA-induced proapoptotic potential.

## Discussion

H_2_S is an emerging novel endogenous neuroprotectant. We have previously demonstrated that H_2_S ablated FA-induced neurotoxicity [26,27]. Emerging evidence support that Brain-derived neurotrophic factor (BDNF) has neuroprotective effect [44–47]. The present work was designed to elucidate whether BDNF is involved in the protection of H_2_S against the neurotoxicity of FA in PC12 cells. Our present study included three significant findings: (1) NaHS, a donor of H_2_S, upregulates the level of BDNF protein in PC12 cells; (2) NaHS markedly rescues FA-induced downregulation of BDNF in PC12 cells; (3) Blocking BDNF-TrkB pathway with K252a, an inhibitor of TrkB receptor, reverses the protective effect of H_2_S on FA-induced neurotoxicity in PC12 cells. Collectively, these results implicate that H_2_S inhibits the neurotoxicity of FA in PC12 cells through upregulating the BDNF-TrkB pathway.

Formaldehyde is the simplest aldehyde that shows high reactivity toward cellular macromolecules like DNA and proteins. Accumulating evidence demonstrate that FA exerts many detrimental effects on the central nervous system (CNS) [5,25,48,49]. FA exposure induces learning and memory impairment, as well as neurotoxicity in vivo and vitro experiments [12,13,26,50–52]. Our previous study shown that FA inhibits the production of endogenous H_2_S [24], and exogenous H_2_S protects PC12 cells against FA-mediated cytotoxicity, apoptosis and endoplasmic reticulum stress [26,27]. However, the exact mechanisms underlying this protective role of H_2_S need to be further studied. Interestingly, our previous study confirmed that H_2_S increases the level of BDNF protein and then attenuates Hcy-induced ER stress and neuronal apoptosis in the hippocampus of rats [38]. Our present findings that NaHS increased the level of BDNF protein and reversed FA-induced down-regulation of BDNF in PC12 cells are consistent with the observation in the hippocampus of rats [38]. BDNF and its receptor TrkB, which are broadly expressed in the CNS, activate various intracellular signaling pathways associated with the neuroprotective effects, including contributions to neuronal survival, synaptic plasticity and cognitive functions [53,54]. Additionally, BDNF plays a prominent role in neuroprotection against a variety of stimuli-induced neuronal cell death, such as oxidative stress and apoptosis [55–57]. Therefore, our present findings that H_2_S upregulates the level of BDNF protein in FA-exposed PC12 cells indicated that BDNF-TrkB pathway may be involved in the protection of H_2_S against FA-induced neurotoxicity and pushed us to confirm whether BDNF mediates the protection of H_2_S against the neurotoxicity of FA in PC12 cells.

In this study, we found that the blockage of BDNF-TrkB pathway with K252a, a specific TrkB receptor inhibitor, reversed the inhibition effect of H_2_S on cytotoxicity of FA, indicating that H_2_S-produced protection against FA-induced cytotoxicity in PC12 cells is mediated by upregulation of BDNF. This is in agreement with the report that upregulation of BDNF prevents human neuronal cells from the cytotoxicity associated with Aβ and H_2_O_2_ [35]. Interestingly, increasing evidence illustrates that oxidative damage is one of the most critical effects of FA exposure [58,59]. Thus, we want to investigate whether inhibition of BDNF-TrkB pathway reverses the protective effect of H_2_S on FA-induced oxidative stress in PC12 cells. We found that treatment with NaHS alone decreases the levels of ROS in PC12 cells. Although it has no statistical significance between NaHS alone group and K252a alone group, we found that K252a revises the NaHS-induced downregulation of ROS levels in PC12 cells. These data imply the potential antioxidant action of H_2_S via regulating BDNF-TrkB pathway. Furthermore, we found that K252a treatment certainty attenuates the inhibitory effect of NaHS against FA-elevated ROS levels in PC12 cells. These data are consistent with the previous finding that BDNF prevent auditory neurons against oxidative damage by significantly down-regulating the levels of ROS and increasing neurons survival [60]. However, the relationship between ROS and pathway of BDNF-TrkB is complex. It has been demonstrated that ROS act in a neuroprotective manner by BDNF-independent activation of TrkB and that the neurotoxic consequences of ROS are paralleled by neuroprotective consequences [36]. Thus, the efficacious therapeutic intervention aimed at diverse CNS disorders is selectively inhibiting the neurotoxic while preserving the nneuroprotective consequences of ROS [36].

Subsequently, we detected the levels of active aldehyde, which is one of the most common products and toxic markers of oxidative stress [61,62]. Malondialdehyde (MDA) and 4-hydroxynonenal (HNE) are two endogenous aldehydes, which are commonly used as a marker of oxidative stress [63]. Similarly, we found that NaHS not only reduced the basic levels of 4-HNE in PC12 cells but also suppressed FA-induced accumulation of 4-HNE and MDA and that K252a application markedly ablates NaHS-induced downregulation of accumulation of MDA and 4-HNE in FA-treated PC12 cells. These results complement our hypothesis that H_2_S protects PC12 cells against FA-induced oxidative stress by upregulation of BDNF-TrkB pathway. Notably, in the present work, we shown that NaHS not completely makes FA-induced upregulation of MDA and 4-HNE levels return to the levels of control group. We have previously confirmed that the neurotoxicity of FA is involved in the disturbed H_2_S synthesis. This implies that it may involve another pathway in FA-induced upregulation of ROS and 4-HNE independent of disturbance in H_2_S generation.

Several studies described that reactive oxygen intermediates and active aldehyde are able to elicit apoptosis in a large variety of cultured cells [64,65]. Then, we further investigated whether inhibition of BDNF-TrkB pathway with K252a abolishes the inhibition of NaHS on FA-induced upregulation of apoptosis and activation of caspase-3, which is a major executioner of apoptosis [66]. Additionally, apoptosis is governed by a number of regulators and the Bcl-2 protein family constitutes a central checkpoint [67]. Change in the levels of poptotic protein Bax and anti-apoptotic protein Bcl-2 is critical for determining cell fate [68]. In this study, we found that K252a treatment reverses NaHS-induced downregulation of Bax protein and upregulation of Bcl-2 protein, indicting the important role of BDNF-Trk B pathway in the protection of H_2_S in PC12 cells. Furthermore, we found that K252a treatment abolishs the prevention of NaHS from FA-induced increases in the apoptotic rate and the activation of caspase-3 in PC12 cells. We also found the K252a reverses the protective effect of H2S against FA-induced upregulation of Bax protein levels and downregulation of Bcl-2 protein levels in PC12 cells. These data suggest that BDNF-TrkB pathway mediates the protective effect of H_2_S against the progression of FA-induced apoptosis in PC12 cells. Taken together, our data indicate that BDNF-TrkB pathway mediates H_2_S-exerted protection against FA-induced neurotoxicity, including cytotoxicity, oxidative stress, and apoptosis in PC12 cells.

Although K252a acts as a specific and potent inhibitor of TrkB, the specificity or lack of specificity of this pharmacological agent should be mentioned. K252a is a potent inhibitor of various protein kinases including Protein kinase A, Protein kinase C and Protein kinase G, while also being a competitive inhibitor with respect to ATP[69]. Because of the lack of specificity of k252a, it remains to be established whether other pathways, such as various protein kinases, mediate H_2_S-induced neuroprotection against FA neurotoxicity. Clearly, in the future, further studies are necessary to understand whether various protein kinases are involved in this neuroprotection of H_2_S.

In summary, the present work identified that H_2_S upregulates the BDNF-TrkB pathway in PC12 cells and that the blockage of BDNF-TrkB pathway reverses the protection of H_2_S against FA-induced cytotoxicity, oxidative stress, and apoptosis in PC12 cells. Our results suggest that the BDNF-TrkB pathway may be a newly contributory mechanism to the protective effects of H_2_S against FA-induced neurotoxicity and other neurotoxicity paradigms. However, further studies are needed to uncover the mechanisms underlying H_2_S-rescued BDNF downregulation. It has been reported sulfhydration involves in various fuctions of H_2_S [70–73] In the future, we will focus on defining whether H_2_S alteres the level of BDNF by sulfhydration.
